# Hybrid control algorithm for flexible needle steering: Demonstration in phantom and human cadaver

**DOI:** 10.1371/journal.pone.0210052

**Published:** 2018-12-31

**Authors:** Navid Shahriari, Janniko R. Georgiadis, Matthijs Oudkerk, Sarthak Misra

**Affiliations:** 1 University of Groningen, University Medical Center Groningen, Center for Medical Imaging-North East Netherlands, Groningen, The Netherlands; 2 Surgical Robotics Laboratory, Department of Biomechanical Engineering, University of Twente, Enschede, The Netherlands; 3 Department of Neuroscience, University of Groningen, University Medical Center Groningen, Groningen, The Netherlands; 4 Department of Biomedical Engineering, University of Groningen, University Medical Centre Groningen, Groningen, The Netherlands; Stanford University, UNITED STATES

## Abstract

Needles are commonly used in the clinic for percutaneous procedures. The outcome of such procedures heavily depends on accurate placement of the needle. There are two main challenges to achieve high accuracy: First, aligning the needle with the targeted lesion, and second, compensating for the deflection of the needle in the tissue. In order to address these challenges, scientists have developed several robotic setups for needle steering. However, the subject is still under research and reliable implementations which can be used in clinical practice are not yet available. In this paper, we have taken some steps in order to bring needle steering closer to practice. A new hybrid control algorithm is developed, which enables us to control a flexible needle by combing base-manipulation and beveled-tip steering methods. A pre-operative path planner is developed which considers the clinical requirements. The proposed method is tested in the lung of a fresh-frozen human cadaver. The work-flow of the experiments are similar to the current clinical practice. Three experimental cases are used to evaluate the proposed steering algorithm. Experimental Case I shows that using the proposed steering algorithm controllability of the needle is increased. In Case II and Case III, the needle is steered in a gelatin phantom and a human cadaver, respectively. The targeting accuracy of 1.35±0.49mm in gelatin phantom and 1.97±0.89mm in cadave is achieved. A feasibility study is performed, in which a fine needle aspiration (FNA) needle is steered in the lungs of a human cadaver under computed tomography guidance. The targeting error for the feasibility study is 2.89±0.22mm. The results suggest that such a robotic system can be beneficial for clinical use and the patient receives less x-ray radiation.

## 1 Introduction

Percutaneous needle insertion is a common minimally invasive surgical procedure used for diagnostic and therapeutic purposes. Lung cancer-related diagnoses and therapies are among the important procedures in the field, due to the high mortality rate worldwide (1.59 million deaths in 2012) [1]. In the United States and Europe lung cancer screening with computed tomography (CT) is recommended for people at high risk or within clinical trial settings [2, 3]. CT-guided lung biopsy is often performed for the nodules greater than 10mm, and for small fast-growing nodules. The CT images are used to locate the lung nodule and then the needle is advanced into the subcutaneous tissue incrementally. A CT scan is acquired after every needle manipulation. This procedure is commonly performed manually by clinicians, and can result in complications such as pneumothorax and pulmonary hemorrhage [4–6]. Core needle biopsy (CNB) or fine needle aspiration (FNA) is used to cut a core for pathological analysis, or to aspirate cell clusters for cytological analysis, respectively. CNB is often reported to result in a higher diagnostic performance, but FNA has a lower complication rate [7, 8].

Although accurate localization of lesions is possible using current imaging technology, those cannot yet be precisely targeted, because of the following issues:

1. The needle tends to deflect from its initial path because of the asymmetric tip.
2. The nodule moves due to physiological motions such as respiration.

**Fig 1:**
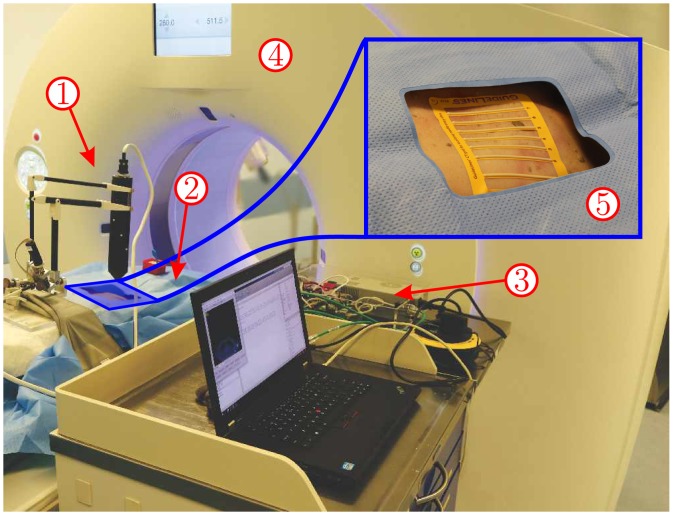
The experimental setup used for robotically steering a fine needle aspiration (FNA) needle in a fresh-frozen human cadaver. The needle is steered towards virtual targets in the lung. Computed tomography (CT) images are used to select the target and the insertion region, and register the target location with respect to the robot reference frame. The experimental setup consists of: ① Needle insertion device. ② Fresh-frozen human cadaver. ③ Control boards. ④ CT scanner. ⑤ Fiducial sticker on the cadaver.

The needle deflection can be used to correct the initial orientation error during the insertion of the needle by rotating the base of the needle. This will not only decrease the amount of needle manipulations, but also enables the clinicians to target even small lung nodules. In order to apply such a solution for steering, robotic setups such the one presented in Fig 1 can be used. In order to address the second issue, breathing instructions are often given to the patient prior to the procedure to minimize the movement. The patient is asked to hold breath in a consistent fashion if the nodule is close to the diaphragm, and therefore, the motion of the nodule will be minimal [9]. Researchers have developed several robotic setups in order to assist clinicians perform needle placement accurately. The literature suggests that such systems can be beneficial for clinical use which are discussed below.

### 1.1 Related work

Different robotic setups, needles and steering algorithms have been developed in order to minimize the lesion targeting error and tissue damage. We discuss some of the relevant approaches below.

#### 1.1.1 Needle steering

The needle steering methods can be categorized by the type of needle that is used. The needles can be either passive (such as symmetric tip [10], bevel-tipped [11], pre-bent/curved tip [12]) or active (such as programmable-bevel [13] and tendon-actuated tip [14]). The advantage of passive needles over active needles is that their design is rather simple. Therefore, those can be used in clinical practice easier and with lower costs. The deflection of bevel-tipped needles (and therefore controllability) is lower than pre-bent/curved tip needles [12]. However, the bevel-tipped needles are currently in use for clinical application, and therefore it is beneficial if the same needle can be used for steering.

Several methods have been developed to steer bevel-tipped needles. Minhas *et al.* introduced the duty-cycling algorithm which allows to control the curvature of the trajectory by spinning the needle [15]. However, the tissue damage is high in this method because of high number of needle rotations. Abayazid *et al.* developed an algorithm that uses the reachable volume of the needle in order to minimize the needle rotations [16]. In this method, the curvature cannot be controlled but the tissue damage is minimal. Relevant literature also showed that symmetric tip needles can be steered by manipulating its base [10, 17, 18]. However, the force applied to the tissue, especially at the insertion point, is high and could tear the tissue. Therefore, we have developed a steering algorithm that introduces minimal tissue damage, while the curvature can be controlled. The algorithm tries to keep the needle on a pre-generated path.

#### 1.1.2 Path planning

Several path planning algorithms have been developed for needle steering purposes. Alterovitz *et al.* developed a 2D path planner using Markov Decision Process (MPD) [19]. This planner prioritized success probability over distance and rotation minimization. Rapidly Exploring Random Trees (RRT) algorithm was used in several studies. Bernardes *et al.* [20] and Patil *et al.* [21] used duty-cycling method with RRT path planning in 2D and 3D environments, respectively. Abayazid *et al.* also used RRT to generate milestones along the optimal path, and steer the needle towards these milestones [16]. The proposed RRT-based algorithms are relatively fast, which makes them suitable for real-time application. The objective in all of these studies were to find a suitable path from the insertion point of the needle to the target. This approach is applicable only if the final configuration of the needle is not of importance. However, depending on the position of the nodule, it is important that the needle reaches the target in a specific configuration in clinical practice. For instance, if the nodule is close to an artery or the heart, the clinicians choose to insert the needle in a direction to maximize the safety of the patient and minimize the chance of hitting the sensitive organs. Therefore, we have developed a pre-operative path planner in which the preferred final direction that the needle enters the nodule, and the insertion region are considered.

### 1.2 Contributions

In this work, we have proposed a new hybrid steering algorithm in which we combine two methods in order to achieve high deflection. In this new algorithm a bevel-tipped needle is steered using the tip steering and a modified version of base-manipulation algorithm. We have used a previously developed CT-compatible robotic system, which has a remote-center-of-motion (RCM). The RCM enables us to apply the developed algorithm. The experiments are performed in a CT scanner in the lungs of a human cadaver in order to validate our method in a completely realistic scenario. This is a key step in bringing needle steering into clinical practice. A pre-operative path planner is developed which provides us the feasible paths to the target, considering the final orientation of the needle and preferred insertion region. We have used clinical FNA needles for the experiments, which show the developed algorithm can be used with common clinical needles, and thus there is no need for specific needle designs.

The paper is organized as follows. Section II describes the developed steering and pre-operative path planning algorithms. The experimental setup, plan and results are presented in section III. Finally, in section IV, we conclude our work and suggest directions for future work.

## 2 Methods

This section presents our hybrid control algorithm which combines tip steering and local-manipulation algorithm. This is followed by describing the pre-operative 3D path planning algorithm.

### 2.1 Hybrid needle deflection model

The deflection of bevel-tipped needles using only tip steering methods is low, especially if thick needles are used. For instance, the radius of curvature for a 22G FNA needle is around 292mm in the breast tissue. Base-manipulation has the advantage of increasing the needle deflection. However, the current approach of the base-manipulation technique causes tissue damage [22]. The shear forces are high at the insertion point because the base of the needle is manipulated and there is no mechanism to minimize the movement of the needle at the insertion point. We present in this section, a new hybrid control algorithm which combines the bevel-tipped needle steering with a modified version of the base-manipulation method. In order to apply the hybrid control, we have developed a needle steering robot with a remote-center-of-motion mechanism. The robot enables us to manipulate the orientation of the needle, while keeping the insertion point fixed. This results in an increase in the deflection of the bevel-tipped needle, while the tissue damage at the insertion point is reduced. The formulation of the new needle manipulation technique, which we have called *local-manipulation* is described below.

**Fig 2:**
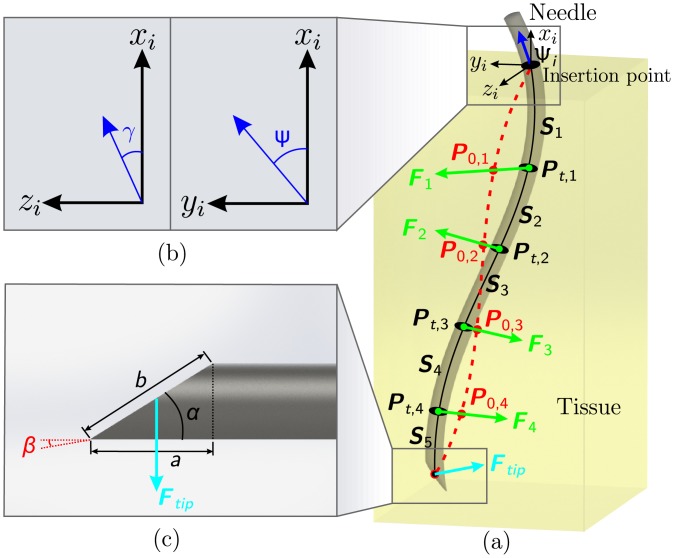
The local-manipulation algorithm, which combines base-manipulation with tip steering is presented. (a) Simplified 3D representation of the local-manipulation algorithm. The needle is inserted into the tissue. Part of the needle which is in the tissue is divided into 5 segments (solid black lines), which are approximated by polynomials ($S_{1}\cdots S_{5}\in R^{3}$). $P_{t,1}\cdots P_{t,4}\in R^{3}$ show the position of the needle points at time *t*. $P_{0,1}\cdots P_{0,4}\in R^{3}$ show the initial position of the needle at positions (1) to (4). Green arrows show the force ($F_{i}\in R^{3\times 1}$) applied to each point on the needle using Eq (5). The light blue arrow represents the force at the tip due to the bevel (***F***_*tip*_) calculated using Eq (8), which is used in tip-steering method. Frame (Ψ_*i*_) depicts the fixed initial frame at the insertion point. The dark blue arrow shows the needle orientation at the insertion point. (b) The needle orientation at the insertion point (blue arrow) is calculated using the inverse kinematics. The orientation is presented using the angles (*γ*) and (*ψ*). These two angles are the inputs for the needle insertion setup. (c) The parameters used to calculate the force and the moment due to the bevel at the tip are presented. Bevel angle is denoted by *α*, and *β* is the cut angle of the needle.

Needle-tissue interaction can be modeled locally using virtual springs placed along the needle shaft [22]. The needle is divided into *N* segments, and a spring is attached to the end of each segment as depicted in Fig 2. This segmentation is performed only for the part of the needle which is inside the tissue. Each segment can be approximated by a 3D polynomial curve ($S_{i}\left(l\right)\in R^{3}$) where *i* ∈ [1, *N*] is the index. Variable (*l* ∈ [0, *L*_*i*_]) is a point on the segment with *L*_*i*_ being the segment arc length. Boundary conditions are applied to the segments to find the polynomial curve coefficients. The second order continuity can be formulated as follows:

$$\begin{array}{c} S_{i}\left(L\right)=S_{i+1}\left(0\right), \end{array}$$ (1)

$${\frac{\text{d}S_{i}}{\text{d}l}|}_{l=L_{i}}={\frac{\text{d}S_{i+1}}{\text{d}l}|}_{l=0},$$ (2)

$${\frac{{\text{d}}^{\text{2}}S_{i}}{\text{d}l^{2}}|}_{l=L_{i}}={\frac{{\text{d}}^{\text{2}}S_{i+1}}{\text{d}l^{2}}|}_{l=0}.$$ (3)

The insertion point of the needle is fixed due to the RCM design of the robot, which results in ***S***_1_(0) = 0. The bending moment at the last element ($M_{tip}\in R^{3\times 1}$) is related to the bevel at the tip and results in

$$\begin{array}{c} M_{tip}={\frac{{\text{d}}^{\text{2}}S_{N}}{\text{d}l^{2}}|}_{l=L_{N}}. \end{array}$$ (4)

The force at the end of each segment ($F_{i}\in R^{3\times 1}$) is directly proportional to the displacement of the needle at that point with respect to its initial position

$$\begin{array}{c} F_{i}=K_{i}\left(P_{t,i}-P_{0,i}\right), \end{array}$$ (5)

where $K_{i}\in R$ is the virtual spring coefficient and depends on the tissue properties. The initial position and position at time (*t*) of the needle in 3D space are denoted by $P_{0,i}\in R^{3\times 1}$ and $P_{t,i}\in R^{3\times 1}$, respectively. Therefore, the shear force along the segment can be written as

$$\begin{array}{c} EI\frac{{\text{d}}^{3}S_{i}}{\text{d}l^{3}}\left(l\right)=-F_{i}. \end{array}$$ (6)

Parameter (*E*) is the needle Young’s modulus and *I* is the second moment of area. For a hollow needle

$$\begin{array}{c} I=\frac{\pi }{64}\left(d_{o}^{4}-d_{i}^{4}\right), \end{array}$$ (7)

where *d*_*i*_ and *d*_*o*_ are inner and outer diameter of the needle.

Misra *et al.* showed that the force and moment applied to the tip of a bevel-tipped needle can be calculated as

$$\begin{array}{c} F_{tip}=\frac{K_{T}a^{2}}{2}\text{tan}\left(\beta \right)-\frac{K_{T}b^{2}}{2}\text{tan}\left(\alpha -\beta \right)\;\text{cos}\left(\alpha \right), \end{array}$$ (8)

$$\begin{array}{c} \begin{array}{cc} M_{tip}= & \frac{K_{T}a^{2}}{6}\text{tan}\left(\beta \right)+\\ & \frac{K_{T}b^{2}}{2}\text{tan}\left(\alpha -\beta \right)(\frac{a}{3}\text{cos}\left(\alpha \right)-\frac{b}{6}\text{sin}{\left(\alpha \right)}^{2}), \end{array} \end{array}$$ (9)

where *K*_*T*_ is the tissue stiffness per unit length [23]. Bevel angle and cut angle are denoted by α∈R and β∈R, respectively. Variables (a∈R) and (b∈R) are related to the tip shape and are shown in Fig 2. The Eqs (8) and (9) can then be substituted in Eqs (6) and (4), respectively. Finally, we have 12 × *N* unknowns and the same number of equations. Therefore, forward or inverse kinematics can be solved as a linear equation. This results in a single control law which includes both the base-manipulation algorithm and also the tip-steering algorithm. In the forward kinematics, the angle of the needle at the insertion point is known and the angle of the tip is calculated. In the inverse kinematics, the desired direction of motion of the tip is known and the corresponding needle angle at the insertion point is calculated.

### 2.2 3D pre-operative path planning

**Fig 3:**
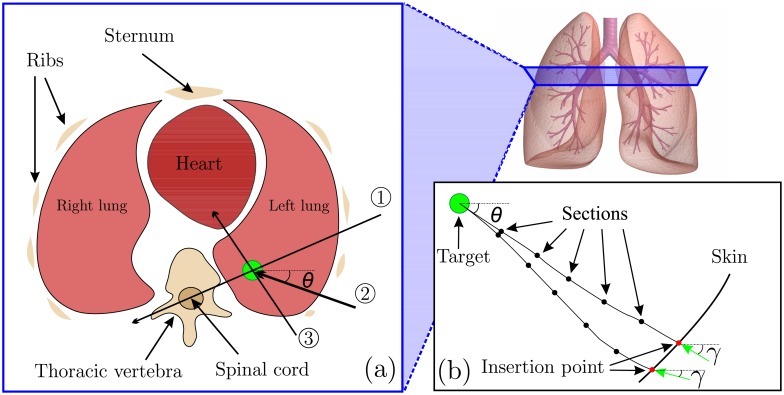
2D representation of the proposed 3D path planner. (a) In a clinical scenario, the direction in which the lesion is approached and the final 3D orientation of the needle are important. ① and ③ show two needle insertion which can be unsafe for the patient because of posterior sensitive organs. However, ② shows a needle insertion path which is relatively safe. Angle (*θ*) represents the final angle of the needle in the target. (b) This inset presents the path planning algorithm principle. The path planner generate all feasible paths starting from the target, and considering the final angle (*θ*). The generated paths are intersected by the skin, and the insertion points and insertion angle (*γ*) are calculated. The green arrows show the needle insertion direction.

The path planning is performed pre-operatively using a 3D scan of the tissue, and the computation time is not critical. Therefore, we have developed an algorithm which can be categorized as a sampling-based path planning. The path planner uses the needle-tissue interaction model to find all the feasible paths to reach the target with the pre-defined orientation and intersect these paths with the surface (skin). In order to generate the paths, the needle trajectory for a single insertion (no rotation) for a defined length is sampled. These points are called sample points. These sample points are generated using the needle-tissue interaction model. The sample points can be rotated in order to achieve all possible trajectories in 3D space. The resolution of samples and rotations are the key factors influencing the time needed to generate the final path. The paths from the target to the surface (skin) are divided into equally spaced section. The number of sections can be set by the user and it depends on the insertion length. Fig 3 shows the algorithm for a simplified 2D case. The needle-tissue interaction model depends on several system parameters, such as tissue stiffness, needle bevel angle and needle Young’s modulus. These parameters are estimated pre-operatively.

## 3 Experiments

This section describes the experiments performed to assess the proposed needle steering and path planning algorithms. The experimental setup and plan are presented below, followed by the results at the end of the section.

### 3.1 Experimental setup

**Fig 4:**
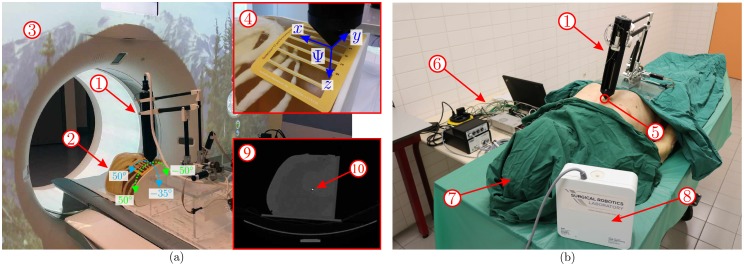
The needle steering is performed using the developed hybrid steering algorithm. The experimental setup for Case II and Case III are depicted in (a) and (b), respectively. ① CT-compatible needle insertion robot with remote-center-of-motion. ② Anthropomorphic gelatin phantom of the thorax. ③ Computed tomography scanner. ④ Sticker fiducial placed on the insertion area, and robot initial reference frame. ⑤ Insertion point. ⑥ Computer and electronics for controlling the robot. ⑦ Fresh-frozen male human cadaver. ⑧ Aurora electromagnetic tracker. ⑨ A CT image of the phantom which demonstrates one of the embedded targets. ⑩ A target embedded in the gelatin phantom.

A CT-compatible robotic setup with RCM resign is used to steer a bevel-tipped needle. The robot has 4 degrees-of-freedom (DOF), which are shown in Fig 4. Two DOFs are used to insert and rotate the needle along its shaft, and the needle can rotate around the insertion point using the remaining two DOFs. The RCM mechanism enables us to apply the hybrid steering algorithm to minimize the tissue damage at the insertion point. Additional details regarding the design and specifications of our setup are provided by Shahriari *et al.* [24, 25].

Two types of needles are used in different experiments. The first needle is Aurora Needle 610062, which is a 2-part needle. It has cannula diameter of 0.83mm (21G) and stylet diameter of 0.55 mm (23.5G), and it is equipped with a 5 DOF EM sensor. In these experiments only the stylet is used in order to achieve more flexibility. An Aurora v3 (Northern Digital Inc., Waterloo, Canada) EM tracker is used to track the sensor. The EM tracker measures the 3D position, and pitch/yaw angles with an accuracy of 0.7mm and 0.2°, respectively. The motor encoder is used to measure the roll angle (rotation about needle axis). The second needle is an FNA spinal biopsy needle with outer diameter of 0.66mm (22G) (Argon Medical Devices, Plano, USA). A Siemens Somatom Force (Siemens AG, Munich, Germany) is used to track the needle and find the target location. The settings are the defaults used for abdomen scan, which are a tube voltage of 90KVP, tube current of 234mAs, pixel spacing of 0.96mm, slice thickness of 0.5mm and convolution kernel of Br40d.

The procedure was performed on a normal-sized male cadaver (died at the age of 73 years) from the Groningen Human Body Donation Program. The body had no lung pathology, and had not undergone surgical procedures on the thorax. After death, the body was shaved. The cadaver was fast frozen to -40°C, then stored at -24°C. Two days prior to the study the body was defrosted to room temperature. The internal ethics committee of the Groningen Human Body Donation Program approved the use of the body.

### 3.2 Experimental plan

Three experimental cases and one feasibility study are conducted to evaluate the proposed algorithms. In all the experiments, the needle is inserted into the tissue with a fixed velocity of 1mm/s.

#### Case I

An FNA needle is inserted into a gelatin phantom using bevel-tip steering and the hybrid steering. The target is placed in an unreachable distance (100mm away from the needle) on x-axis (Fig 4,④) for both methods. This causes the control algorithms to produce the maximum deflection in order to minimize the error. The gelatin phantom is made by mixing 14.9% (by-weight) porcine gelatin powder (Dr. Oetker, Ede, The Netherlands) with 85.1% water. This mixture results in a phantom with a Young’s modulus of 35 kPa, which is similar to breat tissue [26]. Lung tissue is very soft and results in sub-millimeter deflection for the aforementioned needle if bevel-tip steering is used. This results in noisy and not robust curvature estimation. Breast tissue is stiffer than the lung tissue, which could result in more deflection for both methods. This helps to compare the proposed hybrid algorithm with the conventional algorithm. The amount of needle deflection in the two cases are compared. The results are used to validate that hybrid steering can result in a higher deflection of the needle.

#### Case II

**Fig 5:**
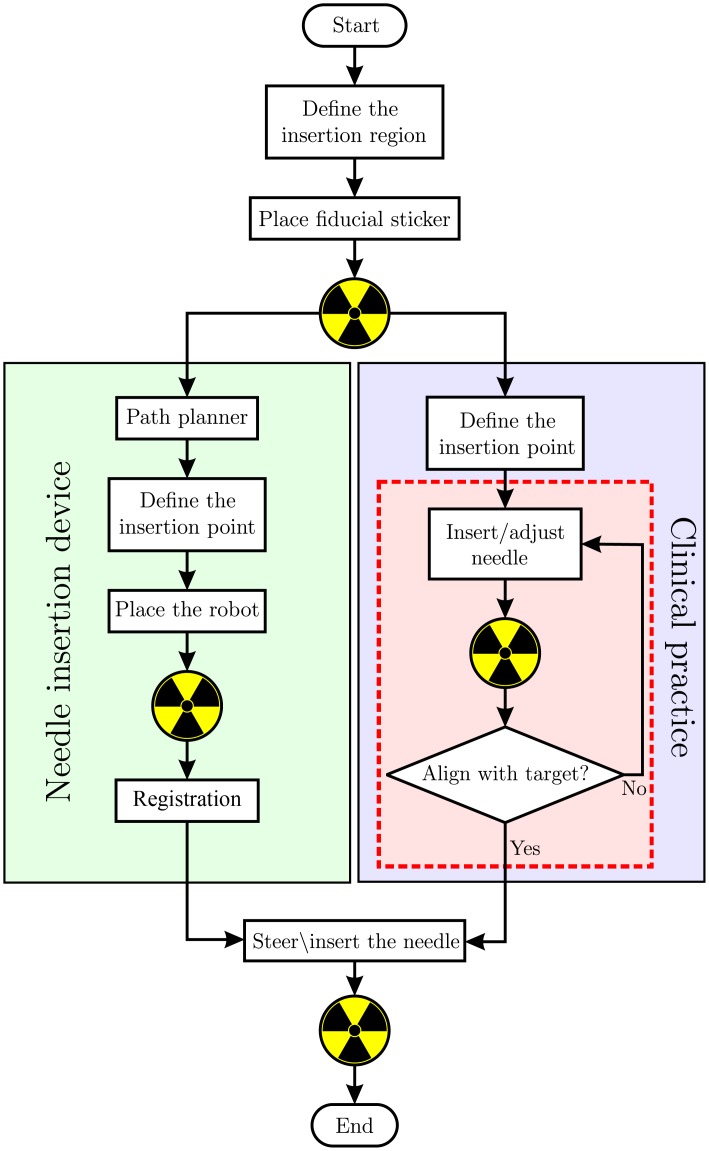
The work-flow of the experiments using the needle insertion device (NID) is as similar as possible to the clinical practice. The radiation symbol (☢) represents a single computed tomography (CT) scan performed. In clinic, aligning the needle with the lesion is challenging. This results in several needle manipulations and CT scans, which is visualized by the red area. This issue is solved using the NID, where the fiducials on the robot are used to calculate the robot pose which aligns the needle with the lesion. The pre-operative path planner provides the insertion point.

The path planning algorithm and the hybrid steering is first tested in an anthropomorphic gelatin phantom of the thorax. The experiments are performed using CT images. An sticker is attached to the phantom, which has 7 cylindrical shape fiducials on it. A CT scan is performed and the fiducials are extracted from the CT images and the skin surface is reconstructed. The target and the final angle is selected and the path planning is executed. The suitable insertion point, and therefore the path, is selected by the user from the feasible option that the path planner provides. Considering the insertion depth, the number of intermediate section of the path planner is set to five. The insertion point is marked using the laser system of the CT scanner and the fiducial sticker. The robot is then placed at the insertion point accordingly, and a new CT scan is performed (Fig 4a). The fiducials on the robot are used to register the robot in the CT scanner reference frame. The insertion is then performed automatically and a final CT scan is taken to check the error. The work-flow of the experiment is depicted in Fig 5.

#### Case III

A needle equipped with an electromagnetic (EM) sensor is steered towards a virtual target in fresh-frozen human cadaver. In this experiment, the needle is tracked in real-time using an EM tracker, and the needle controlled using the hybrid steering method. In this case, the skin is perforated using a surgical scalpel blade, in order to minimize skin influence on the needle trajectory and to avoid damaging of the needle’s tip. The insertion point is altered for each insertion. The experimental setup is presented in Fig 4. Experimental Case II and Case III are summarized in Table 1.

**Table 1:** The details of the needle steering experimental Case II and Case III.

	Needle	Target	Tracking	Path planner	Tissue
**Case II**	Fine needle aspiration	Real	Computed tomography	✓	Gelatin
**Case III**	Electromagnetic	Virtual	Electromagnetic	✕	Cadaver

#### Feasibility study

In this study, we try to resemble the clinical conditions to evaluate our system in a realistic setup. A fresh-frozen human cadaver is used to target two virtual lesions in the lungs with an FNA needle. The work-flow is similar to Case II, which is usually used for lung/liver biopsies. The experimental setup for this study is shown in Fig 1. The skin is perforated using a surgical scalpel blade in this case as well.

### 3.3 Results

**Fig 6:**
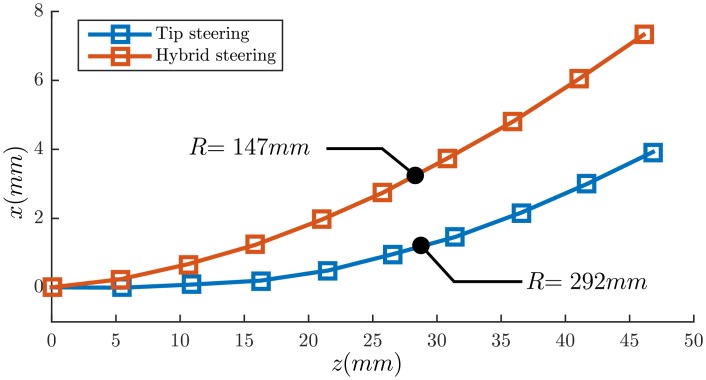
Experimental Case I: The needle is steered using tip and hybrid steering algorithms. This figure demonstrates a representative measurement for tip steering (blue) and hybrid steering (red). The axis *x* and *z* are according to the coordinate system shown in Fig 4. The data points demonstrated with square shapes are the needle tip positioned measured using an electromagnetic tracker. The solid lines are arcs with corresponding radius of curvature. The radius of curvature (*R*) shows a decrease when using the hybrid control, which results in higher controllability of needle trajectory with respect to tip steering. The steering is performed 5 times for each method, which results in radius of curvature of 289.6 ± 7.2*mm* and 155.8 ± 8.7*mm* for tip and hybrid steering, respectively.

**Fig 7:**
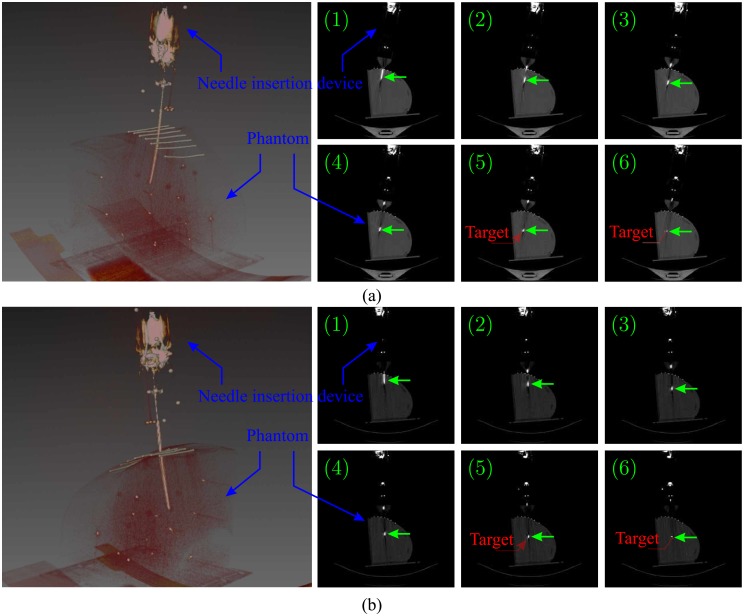
Representative results for experimental Case II: The needle trajectory of two experiments in the anthropomorphic gelatin phantom of the thorax. On the left are the 3D visualization of the experiments. On the right are the CT images showing the needle (green arrow) trajectory in several consecutive slices. The location of the real target is highlighted by the red circle/arrow.

**Fig 8:**
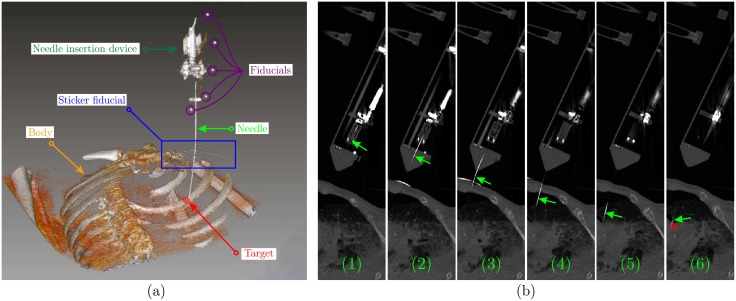
Feasibility study: An FNA needle is steered towards 2 virtual targets in a fresh-frozen human cadaver. (a) The 3D visualization of the body, the needle, the needle insertion device and the fiducial sticker. The insertion point is selected so that the needle enters the chest in-between the ribs. (b) The CT images showing the needle (green arrow) trajectory in several consecutive slices. The location of the virtual target is indicated by the red circle.

In the experimental Case I, the tip steering is compared with hybrid steering. Each method is repeated 5 times and the radius of curvature is measured. The radius of curvature is 289.6 ± 7.2*mm* for the tip steering and 155.8 ± 8.7*mm* for the hybrid steering. This means around 17mm of deflection in 10cm of insertion in comparison with approximately 39mm of deflection. This results show that hybrid steering can improve the amount of deflection and therefore the controlability. The results are presented in Fig 6. In experimental Case II, the needle is steered towards 5 real targets. The targets are spheres with a radius ranging between 3mm to 8mm, and these are placed randomly in the phantom. The needle is steered towards the center of the targets. The error is calculated as the absolute distance between the target and needle tip position in 3D space, and also the final angular error. The error is calculated by performing a final CT scan. The mean targeting error is 1.35±0.49mm. The results are presented in Table 2 and Fig 7. In experimental Case III, the targets are virtual points in 3D space within the cadaver lung, and the locations are chosen randomly. The steering experiments are repeated 5 times. The mean targeting error is 1.97±0.89mm and the results are available in Table 2. The feasibility study is performed twice and the needle is steered towards virtual targets within the lung. A reconstructed 3D visualization of the second feasibiliy study is shown in Fig 8(a). The needle trajectory for the same experiments is shown in Fig 8(b), where the needle is pointed by the green arrows. The mean targeting error for the feasibility study is 2.89±0.22mm. The radius of curvature for the two insertions are 223.74mm and 184.57mm, respectively. The radius of curvature is calculated based on the first 25mm section of the needle, where the effect of hybrid steering is better visible. It is worth mentioning that the radius of curvature is not defined for the needle as a whole, since the trajectory is in 3D space and curvature could only be calculated locally.

**Table 2:** Experimental results: The results of the experimental Case II and Case III are presented. The target location, and error for each case are indicated. The error is calculated as the absolute distance between the tip of the needle and the center of the target. The mean and standard deviation of the error for each experimental scenario are presented separately. Radius of curvature (R) is calculated for the first 25mm of insertion. The angular error is the final needle orientation error with respect to the angle set in the path planner.

Experiments	#	Target x (mm)	Target y (mm)	Target z (mm)	Error (mm)	R (mm)	Mean error (mm)	Angular error (deg)
**Case II**	1	−8.57	14.64	44.69	1.05	196.62	1.35 ± 0.49	3.48
	2	−7.86	20.92	61.14	1.59	240.60		0.98
	3	12.23	26.70	64.19	1.94	255.58		7.12
	4	−1.01	25.89	70.24	0.54	162.00		2.88
	5	12.70	27.49	66.04	1.66	198.28		5.42
**Case III**	1	−3.50	1.00	50.00	2.67	N/A	1.97 ± 0.89	N/A
	2	1.50	−6.50	60.00	0.83			
	3	−2.00	−3.00	60.00	2.27			
	4	6.00	3.00	60.00	1.25			
	5	−2.00	−4.00	70.00	2.87			

## 4 Discussion and future work

In this study, we have presented a new algorithm to steer a flexible needle by combining tip-based control with the base-manipulation control. The algorithm is developed for a CT-compatible needle insertion setup, which has a remote-center-of-motion at the point of insertion. The combination of the robotic setup and the developed control algorithm results in an increase in the deflection of the needle. A pre-operative path planner is developed, in which we consider the location of the target, the required final pose of the needle and the preferred insertion region. The path planner provides the feasible paths and the surgeon can choose the preferred path.

### 4.1 Discussion

The needle steering algorithm, along with the path planner, are tested using 3 experimental cases which are discussed below. The results of the first experimental case shows that the needle deflection has increased using the hybrid control with respect to the tip-steering. This results in higher controlability of the needle trajectory. It is important to mention that the deflection depends on the amount of base rotation of the needle. Therefore, the deflection can go even higher, but the disadvantage is higher tissue damage. In these experiments we have set a limit of 20° on the rotation of the needle at the insertion point. In the experimental Case II, the needle is steered towards real target in a an anthropomorphic gelatin phantom of the thorax. The targeting accuracy is higher than the case with human cadaver, which is due to homogeneity of the gelatin phantom. The mechanical properties of the gelatin phantom is known, and therefore the model used for steering is accurate. In experimental Case III, the tip of the needle is tracked in real-time using the EM tracker. Therefore, the control loop is closed using the measurements, which in principle increase the targeting accuracy. However, the needle is steered in a human cadaver which is not homogeneous. To reach the target in the lung, the needle has to pierce the following layers of the thoracic wall: muscle fascia, external intercostal muscle, internal intercostal muscle, innermost intercostal muscle, endothoracic fascia, parietal pleura, visceral pleura. The mechanical properties of these layers are different and these are not accurately available. This results in a lower targeting accuracy with higher standard deviation. In the feasibility test, the robot was positioned over the left 2nd intercostal space. The space between the parietal and visceral pleural usually contains just a film of fluid, but with defects of the thoracic wall (e.g. by piercing or cutting it) air can fill this space and push the lung away (pneumothorax). Indeed, the CT-experiment showed that the pleural space was wider than normal, possibly due to earlier experiments/punctures performed on the cadaver. This resulted in higher targeting error. The authors have showed in their previous study [25] that fusing the real-time EM tracking data with CT images can increase the targeting accuracy. However, in clinic, the procedures should be made as straight forward as possible for the clinicians, as far as the procedure could be performed with the required accuracy. In this study, it was shown that even in absence of data fusion of CT images and a real-time sensing device, an accuracy of around 3mm could be achieved in human tissue. The smallest lung nodules which physicians usually try to reach with a needle, assuming a spherical shape, is about 5mm diameter. This is very challenging even for experienced physicians and it involves several iterations of insertions, retraction and manipulation of the needle. If the center of the nodule is selected as the target, a robotic system such as the one presented here can reach targets of 6mm or larger in one step, without the need of retraction and re-insertion of the needle. Furthermore, the measure of the radius of curvature for Case II and the Feasibility Study shows that the hybrid control is effective and is used during all the insertion, in the sense that higher deflection is realized. It demonstrates that even in the case of insertions into the lung (softer tissue), still the radius of curvature is smaller than bevel-tip steering in gelatin (stiffer tissue).

### 4.2 Future work

In this work, we took some steps in order to use needle steering in a clinically relevant situation. We have used a work-flow similar to the protocol which is used in clinical practice. However, further experiments are needed before the setup can be used for patient studies. In the future, we want to perform more human cadaver experiments using the CT scanner, not only to validate the accuracy of the system, but also to evaluate the design and user-friendliness of the setup. Furthermore, we are planning to perform several experiments on live animals in order to check the effects of biological motions on the needle steering accuracy and trying to compensate for the motion.
